# Smartphone addiction among university students in Tunisia

**DOI:** 10.1192/j.eurpsy.2023.1361

**Published:** 2023-07-19

**Authors:** M. Turki, H. E. Mhiri, F. jmil, A. Samet, N. Halouani, S. Ellouze, J. Aloulou

**Affiliations:** Psychiatry “B”, Hedi Chaker University Hospital, Sfax, Tunisia

## Abstract

**Introduction:**

Popularity and availability of smartphones have dramatically increased in the past years, and have led to a great impact on people’s daily lives changing their habits and behaviors. This trend is accompanied by increased concerns regarding potentially adverse effects of problematic smartphone use, particularly with respect to physical and mental health.

**Objectives:**

In this study, we investigated prevalence and associated factors of smartphone addiction among Tunisian university students.

**Methods:**

It was a cross-sectional, descriptive and analytical web-based study, conducted among 144 university students in Tunisia. Data were collected using an online questionnaire spread throughout social media (Facebook), using the Google Forms® platform, during September and October 2022. We used the “Smartphone Addiction Scale-Short Version” (SAS-SV).

**Results:**

The mean age of participants was 23.38±3.27 years, with a sex-ratio of (F/M) of 2.8.

Tobacco, alcohol and cannabis use was noted respectively in 12.5%, 3.5% and 3.5% of cases.

The mean score SAS-SV was 37.92±8.82. Among the students, 68.8% were considered at high risk of Smartphone addiction.

Male students were more likely to be at higher risk of smartphone addiction than females, without a significant relationship.

Scores of SAS-SV were significantly higher among cannabis users (48.4 vs 37.54; p=0.006) and non-medical students (39.07 vs 36.11; p=0.049)

**Conclusions:**

University students are a particularly vulnerable population to smartphone addiction, and this may lead to negative psychosocial effects. Educational awareness and preventive measures should be implemented.

**Disclosure of Interest:**

None Declared

